# Comparative impact of tertiary lymphoid structures and tumor-infiltrating lymphocytes in cholangiocarcinoma

**DOI:** 10.1136/jitc-2024-010173

**Published:** 2025-01-27

**Authors:** Shin-Yi Chung, Yi-Chen Yeh, Chien-Jung Huang, Nai-Jung Chiang, Dennis Shin-Shian Hsu, Ming-Hsien Chan, Meng-Lun Lu, Tzu-Sheng Hsu, Yi-Ping Hung, Chun-Nan Yeh, Michael Hsiao, Yu-Chan Chang, Yu-Chao Wang, Ming-Huang Chen

**Affiliations:** 1 Department of Oncology, Taipei Veterans General Hospital, Taipei, Taiwan; 2 Institute of Biomedical Informatics, National Yang Ming Chiao Tung University, Taipei, Taiwan; 3 Department of Pathology and Laboratory Medicine, Taipei Veterans General Hospital, Taipei, Taiwan; 4 School of Medicine, National Yang Ming Chiao Tung University, Taipei, Taiwan; 5 National Institute of Cancer Research, National Health Research Institutes, Tainan, Taiwan; 6 Asclepiumm Taiwan Co., Ltd, Taipei, Taiwan; 7 Department of Biomedical Imaging and Radiological Sciences, National Yang-Ming University, Taipei, Taiwan; 8 Institute of Molecular and Cellular Biology, College of Life Sciences and Medicine, National Tsing Hua University, Hsinchu, Taiwan; 9 General Surgery, Chang Gung Memorial Hospital, Taoyuan, Taiwan; 10 Genomics Research Center, Academia Sinica, Taipei, Taiwan

**Keywords:** Tumor Lysis Syndrome - TLS, Tumor infiltrating lymphocyte - TIL, Tumor microenvironment - TME, Immunotherapy

## Abstract

**Background:**

Cholangiocarcinoma is a challenging malignancy with limited responses to conventional therapies, particularly immune checkpoint inhibitor therapy. Tumor-infiltrating lymphocytes (TILs) and tertiary lymphoid structures (TLSs) are key components of the tumor microenvironment (TME) and have been implicated in the immune response to cancer. However, the role and difference of TLSs and TILs in patients with cholangiocarcinoma remains unclear. This study elucidates their contributions to the TME.

**Methods:**

We examined 16 tumor samples from a single-arm, phase II trial of nivolumab plus modified gemcitabine and S-1 and various datasets. Immunohistochemistry and RNA sequencing were employed to assess TLSs and TILs presence and activity. Differential gene expression and signature of immune cell composition were examined by GeoMx Digital Spatial Profiler and Cancer Transcriptome Altas analysis.

**Results:**

TLS-positive (N=7) patients demonstrated significantly better immunotherapy outcomes compared with TLS-negative (N=9) patients, including higher objective response rates (71% vs 0%) and disease control rates (100% vs 67%). The presence of TLSs correlated with improved progression-free and overall survival (p=0.03). TLSs were associated with “inflamed” tumors characterized by substantial immune infiltration, particularly involving T and B cells. Gene expression analyses identified significant upregulation of B cell-related genes in TLSs. Additionally, TLSs exhibited higher properties of memory B cells and myeloid dendritic cells but lower levels of innate immune cells compared with TILs. T cells within TLSs showed elevated expression of precursor-exhausted-related genes and lower cytotoxicity signature. Furthermore, TILs in TLS-positive tumors had higher levels of exhaustion signatures compared with TILs in TLS-negative tumors. Clinical data corroborated these findings, with higher PD-L1 and LAG-3 expression in TLS-positive tumors.

**Conclusion:**

Our findings revealed that TILs in TLS-positive tumors have more exhausted T cell signature and PD-1 and LAG-3 protein expression in CCA which support our clinical finding. TLSs can predict favorable immunotherapy responses in patients with cholangiocarcinoma, highlighting their potential as a biomarker and therapeutic target to enhance treatment efficacy.

**WHAT IS ALREADY KNOWN ON THIS TOPIC**

- Cholangiocarcinoma presents substantial treatment challenges, and its tumor microenvironment, rich in tumor-infiltrating lymphocytes (TILs) and tertiary lymphoid structures (TLSs), plays a crucial role in response to immunotherapy. However, the distinct roles and impacts of TLSs and TILs in cholangiocarcinoma outcomes were previously unclear, warranting further investigation.

**WHAT THIS STUDY ADDS**

- This study reveals that TLS-positive patients with cholangiocarcinoma show significantly improved responses to immunotherapy, including higher rates of objective response and disease control, compared with TLS-negative patients. It also uncovers unique gene expression profiles in TILs and TLSs, highlighting the influence of TLSs on immune cell exhaustion and immune checkpoint molecule expression.

**HOW THIS STUDY MIGHT AFFECT RESEARCH, PRACTICE OR POLICY**

- The findings suggest that TLSs could serve as valuable predictive biomarkers for immunotherapy efficacy in cholangiocarcinoma, offering a potential target to optimize treatment strategies. Further research may focus on leveraging TLS presence to enhance immunotherapy responses in this challenging malignancy.

## Introduction

 Cholangiocarcinoma, a malignancy arising from the biliary epithelium, presents therapeutic challenges because of its complex interplay with the immune microenvironment.^1 2^ On the basis of the lesion location, cholangiocarcinoma tumors can be classified as extracellular, intrahepatic, or peripheral tumors. These types differ in terms of their characteristics and immune signatures. The standard treatment strategies for cholangiocarcinoma include chemotherapy, radiation therapy, and targeted therapy.^3^ Recently, a combination of immunotherapy and chemotherapy has emerged as the standard treatment strategy for this condition.^4^,^6^ The identification of parameters for analyzing tertiary lymphoid structures (TLSs) or tumor-infiltrating lymphocytes (TILs) would enable the objective evaluation of the efficacy of cholangiocarcinoma treatment. Our previous study revealed favorable treatment responses and survival in patients receiving nivolumab plus modified gemcitabine and S-1 therapy for advanced cholangiocarcinoma.^7^ The tumor microenvironment (TME) is pivotal in regulating immune responses, either through suppression or enhancement, leading to the classification of tumors as inflamed (or called hot) or non-inflamed (or called cold). Inflamed tumors are distinguished by robust inflammatory activity, reflecting significant immune cell infiltration within the tumor tissue. In contrast, non-inflamed tumors exhibit minimal immune cell infiltration, indicative of a deficient or impaired immune response. This distinction has important implications for understanding tumor immunity and the effectiveness of immunotherapeutic strategies.^8^,^10^ Favorable immunotherapy responses are dependent on dynamic interactions between immunomodulators and tumor cells in the TME.^11^

Immunotherapy rejuvenates cytotoxic T cells to fight cancer. However, only 20% of patients receiving immunotherapy exhibit lasting benefits.^12^ TILs, which are frequently detected in the tumor stroma and core,^13^ target and eliminate tumor cells; their presence within tumors is commonly associated with improved clinical outcomes after surgery or immunotherapy.^14^,^16^ Immune diversity within solid tumors necessitates the use of TILs with varying specificities; consequently, TIL-based treatment has emerged as safe personalized immunotherapy.^17 18^ Furthermore, research has revealed additional elements within the TME that potentially affect the treatment response; these elements include myeloid cells and diverse immune cell subsets.^19^ TLSs are structures rich in T cells, dendritic cells (DCs), and B cells, particularly follicular B cells with germinal centers.^1220^,^22^ Increasing research has revealed strong correlations between tumor-associated TLSs and favorable clinical outcomes across cancers, including colorectal cancer, hepatocellular carcinoma, melanoma, and non-small cell lung cancer.^23^,^25^ Evidence suggests that B-cell populations within TLSs play major roles in improving the immunotherapy response and survival.^26^,^28^

Suitable predictors of patient responses to various cancer therapy modalities are urgently required; this urgent requirement necessitates research for identifying TILs’ distinct gene signatures and immune cell compositions and for clarifying their roles in shaping the immune landscape of tumors. Increasing attention has been paid to TIL–TLS interactions, which may predict disease progression and the treatment response.

In this study, we explored the distinct gene signatures and immune cell compositions of TILs and TLSs present in the cholangiocarcinoma microenvironment. In addition, we examined the TIL and TLS profiles of patients with cholangiocarcinoma receiving nivolumab plus modified gemcitabine and S-1 therapy.^7^ Notably, TLS-positive patients exhibited markedly improved immunotherapy responses, emphasizing the potential of TLSs for predicting clinical outcomes in patients with cholangiocarcinoma.

## Methods

### Patients

The present study included patients who had received a histologically confirmed diagnosis of locally advanced or metastatic biliary tract cancer at any of the participating hospitals (assessed on the basis of the Response Evaluation Criteria in Solid Tumors (V.1.1)). Other clinical data were obtained from a relevant study.^7^ The patients received fixed-dose therapy (nivolumab, 200 mg; gemcitabine, 800 mg/m^2^; and S-1, 80/100/120 mg) on day 1 of a 2-week cycle. S-1 was continued until the occurrence of any adverse event or the need for dose reduction (as determined by the investigators). Treatment cycles were continued until the progression of the disease, the occurrence of intolerable toxic reactions, withdrawal of consent, or other reasons. Subsequent cycles were initiated only for patients satisfying specific criteria on day 1. Dose adjustment cannot be conducted for nivolumab. However, the doses of gemcitabine and S-1 can be reduced twice—to minimum doses of 400 mg/m^2^ and 60 mg/day, respectively—without further escalation.

### Data collection and analysis

To study inflamed and non-inflamed tumors in patients with cholangiocarcinoma, we analyzed the RNA-Seq data of cholangiocarcinoma samples. The data were downloaded from The Cancer Genome Atlas (TCGA; https://portal.gdc.cancer.gov/) and the Gene Expression Omnibus (RRID:SCR_005012; https://www.ncbi.nlm.nih.gov/geo/) databases. A TCGA data set—TCGA-cholangiocarcinoma (TCGA-CHOL; n=36)—was used for discovery, whereas three GEO data sets, all of which consist of intrahepatic cholangiocarcinoma (ICC) cases, GSE119336 (n=15), GSE162396 (n=12), and GSE215997 (n=13), were used for validation. For the transcriptomic analysis of ICC, three GEO datasets (GSE119336, GSE162396, and GSE215997) were used. GSE119336 and GSE162396 provided RNA-Seq and gene expression data from ICC tumors and matched normal tissues. GSE215997 contained transcriptomic profiles of cholangiocarcinoma and adjacent normal tissues. Raw reads data (FASTQ files) were downloaded from GEO and processed using standard bioinformatic workflows, including quality control, alignment and normalization. We used microenvironmental cell population (MCP)-counter^29^ to quantify the abundance (MCP scores) of eight immune cells (T cells, CD8^+^ T cells, cytotoxic lymphocytes, natural killer (NK) cells, B-cell lineages, monocytic lineage, myeloid DCs, and neutrophils) and two stromal cells (endothelial cells and fibroblasts). Gene expression data (fragments per kilobase million values) were log2-transformed. MCP scores were calculated using 109 transcriptomic markers. Cholangiocarcinoma samples were stratified into inflamed and non-inflamed tumors based on MCP scores through hierarchical clustering (distance metric: Euclidean distance; linkage criterion: Ward method). On the basis of MCP score heatmaps and hierarchical clustering results, we defined samples with high and low MCP scores across cell types as inflamed and non-inflamed tumors, respectively. Furthermore, principal component analysis (PCA) was conducted on 109 transcriptomic marker genes expression to identify the predominant genes for classification. A pathologist (Y-CY) reviewed whole-slide images from the TCGA-CHOL data set to detect the presence of TLSs in inflamed and non-inflamed tumors.

The profiles and relevance of cholangiocarcinoma cell lines were assessed using data from the Cancer Cell Line Encyclopedia and Depmap portal website (https://depmap.org/portal/ccle/). Customized statistics for each site were evaluated to obtain significant data (p≤0.05).

### Next-generation sequencing

To analyze the genetic differences in each sample, tumors were collected and performed the 440-gene panel ACTOnco from ACT Genomics, with sequencing carried out on the Ion Torrent platform by Thermo Fisher Scientific. The methodology followed was as previously outlined.^7^ All samples were sequenced with an average mean depth of ≥500×, ensuring that at least 75% of sequenced regions had coverage at ≥100×.

### Immunohistochemistry

For immunohistochemical analysis, 4 μm thick formalin-fixed, paraffin-embedded cholangiocarcinoma tissue specimens were stained (overnight at 4°C) with primary antibodies against CD4, CD3, CD8, CD21, MS4A1 (CD20), and CD79A. Simultaneously, control specimens were incubated in diluent without primary antibody. After incubation, the slides were washed thrice (5 min/wash) with Tris-buffered saline containing 0.1% Tween 20 and then visualized using the Real Envision Detection System, Peroxidase/DAB+, Rabbit/Mouse (K500711; Dako). After washing, the slides were counterstained with hematoxylin and then analyzed through microscopy by a pathologist in a blinded manner.

### H&E staining

Tissue samples from each patient with cholangiocarcinoma were sectioned and fixed in 10% formalin; this was followed by dehydration in graded alcohols and embedding in paraffin wax. Subsequently, 0.2 μm thick sections were sliced from the paraffinized blocks; the sections were deparaffinized through immersion in xylene and rehydrated. H&E was added to each slide, which was then rinsed with water. Next, each slide was dehydrated through immersion in graded alcohols and then in xylene (twice). Photomicrographs were obtained and interpreted by the pathologist (Y-CY). TLSs and TIL evaluation was performed in H&E-stained slides. TLS was defined as dense cellular lymphoid aggregates resembling germinal centers found in secondary lymphoid structures.^30^ TIL was defined as lymphocytes infiltrating tumor nests or located dispersed in the stroma between the carcinoma cells.^31^

### Spatial transcriptomic data acquisition

Spatial transcriptomic data were generated using the GeoMx Digital Spatial Profiling (DSP) platform (NanoString Technologies), which enables the collection of spatially resolved gene expression data from formalin-fixed paraffin-embedded tissue sections. Tissue sections were stained with up to four visualization markers, and regions of interest (ROIs) were selected based on cell type-specific markers, allowing the profiling of tumor and microenvironment regions. To perform spatial analyses in the present study, we analyzed six cholangiocarcinoma samples derived from our study: three TLS-positive and three TLS-negative tumor tissue sections. Within each section, 12 ROIs were selected on the basis of visualization markers: CD19 (yellow) for B cells, CD3E (red) for T cells, KRT18 (green) for tumor cells, and DNA (blue) for nuclei. Each ROI only contained a single area of illumination (AOI) or segmentation, ensuring focused analysis of specific regions.

### Single-cell RNA sequencing data

To define reference cell-type profiles, single-cell RNA sequencing (scRNA-seq) data from publicly available datasets were used. These datasets provided detailed profiles of immune and stromal cell populations, which were then curated to create a cell profile matrix. The matrix was constructed from a combination of flow-sorted peripheral blood mononuclear cells, scRNA-seq data from tumors, and RNA-seq data from flow-sorted stromal cells, collectively forming the SafeTME matrix. Deconvolution was performed using the SpatialDecon algorithm, which applies log-normal regression to spatial transcriptomic data to estimate the abundance of immune cell populations. The algorithm incorporates reference cell-type profiles from the SafeTME matrix to quantify the presence of 18 immune and stromal cell types within each ROI. The log-normal regression model corrects for variability in gene expression data, providing more accurate cell type estimates compared with classical least-squares methods. To ensure consistent and comparable gene expression measurements across samples, Q3 normalization was applied to the GeoMx DSP data. In this method, the 75th percentile (third quartile, Q3) of the signal from each ROI was calculated to account for technical variations across regions. This approach normalizes the data by adjusting each ROI’s gene expression values relative to its 75th percentile, thereby minimizing the impact of outliers or highly expressed genes. The Q3 normalized data were then used for downstream analyses, including differential gene expression and cell type deconvolution.

### Geomx Cancer Transcriptome Atlas Profiling

Tissue samples were profiled using the GeoMx Cancer Transcriptome Atlas (CTA) from NanoString Technologies, which provides a comprehensive spatial transcriptomic analysis across a variety of cancer types. The CTA enables the measurement of over 1800 genes specifically associated with key biological processes such as the TME, immune response, stromal interactions, and cancer progression. These genes cover a wide spectrum of pathways involved in tumor development, immune modulation, and therapeutic resistance.

### Statistical analysis

Data are presented as mean±SD. Between-group differences were determined using the Mann-Whitney U test. The rates of progression-free survival and overall survival were calculated using the Kaplan-Meier method. Several clinicopathological factors were considered in the initial univariate analysis, which was performed using a log-rank test. Statistical analyses were performed using SPSS (V.17.0) for Windows (IBM SPSS).

## Results

### TLSs can predict immunotherapy responses in patients with cholangiocarcinoma

**Figure 1:**
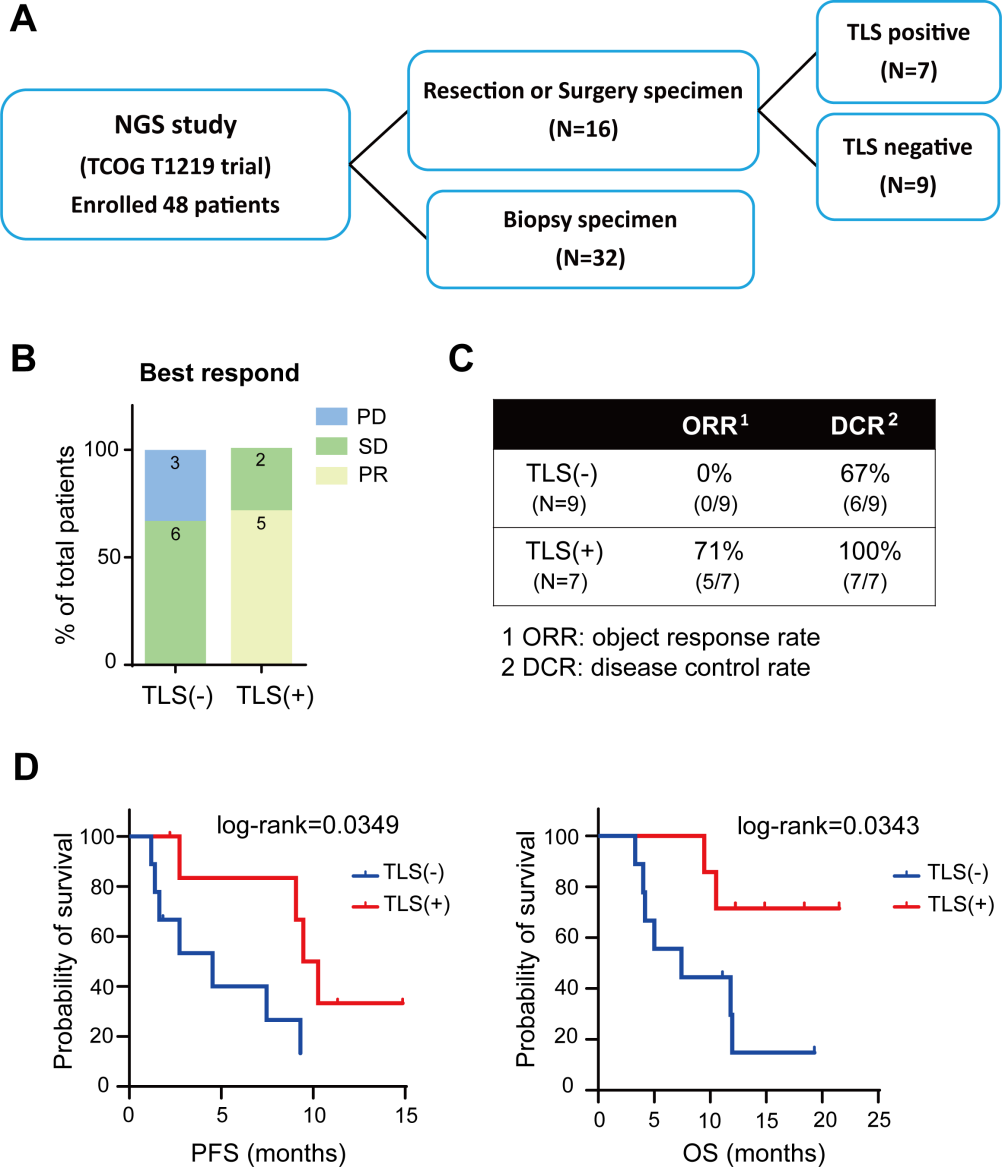
Clinical response of TLS-positive or TLS-negative patients with cholangiocarcinoma (CCA) receiving immune checkpoint inhibitors. (**A**) Flow chart depicting the selection of suitable samples from the TCOG1219 data set. (**B**) Optimal immunotherapy response in each TLS-positive or TLS-negative patient was identified through H&E staining. Kaplan-Meier curves for (**C**) progression-free survival (PFS) and (**D**) overall survival (OS) in TLS-positive or TLS-negative patients (p=0.03 and 0.03, respectively). (**E**) Genomic landscape of the most common somatic mutation in 14 CCA patients, 7 with TLS positive and seven without TLS. CR, complete response; DCR, disease control rates; ORR, objective response rate; PD, progressive disease; PR, partial response; SD, stable disease; TLSs, tertiary lymphoid structures.

To investigate whether TLSs can predict immunotherapy responses in patients with cholangiocarcinoma, the tumor samples derived from our clinical study—a single-arm, phase II trial investigating the potential of nivolumab plus modified gemcitabine and S-1 therapy as a first-line treatment modality. Given that TLS is organized with distinct T and B cell zones and follicular DCs,^12^ a total of 32 biopsy and 16 resection/surgery samples were analyzed by H&E staining to identify TLS. The numbers of TLS-positive and TLS-negative samples were 7 and 9, respectively (figure 1A). Compared with TLS-negative patients, TLS-positive patients exhibited significantly improved immunotherapy responses, which were characterized by substantial increases in both the objective response rate (complete response+partial response; 0% vs 71%, respectively) and disease control rate (complete response+partial response+stable disease; 67% vs 100%, respectively; figure 1B). Furthermore, the rates of progression-free survival and overall survival were higher in TLS-positive patients than in TLS-negative patients (p=0.03 for both; figure 1C,D). Interestingly, to investigate whether the formation of TLS is related to different oncogene mutations, we sorted out the most common frequency mutation genes in CCA (Cholangiocarcinoma) to compare in TLS-positive and TLS-negative patients. Our results observed that the formation of TLS is not strongly related to any different oncogene mutations (figure 1E). Collectively, our results support the potential of TLSs for predicting the outcomes of combined immunotherapy in patients with cholangiocarcinoma.

### Classification of cholangiocarcinoma samples as inflamed or non-inflamed tumors

**Figure 2:**
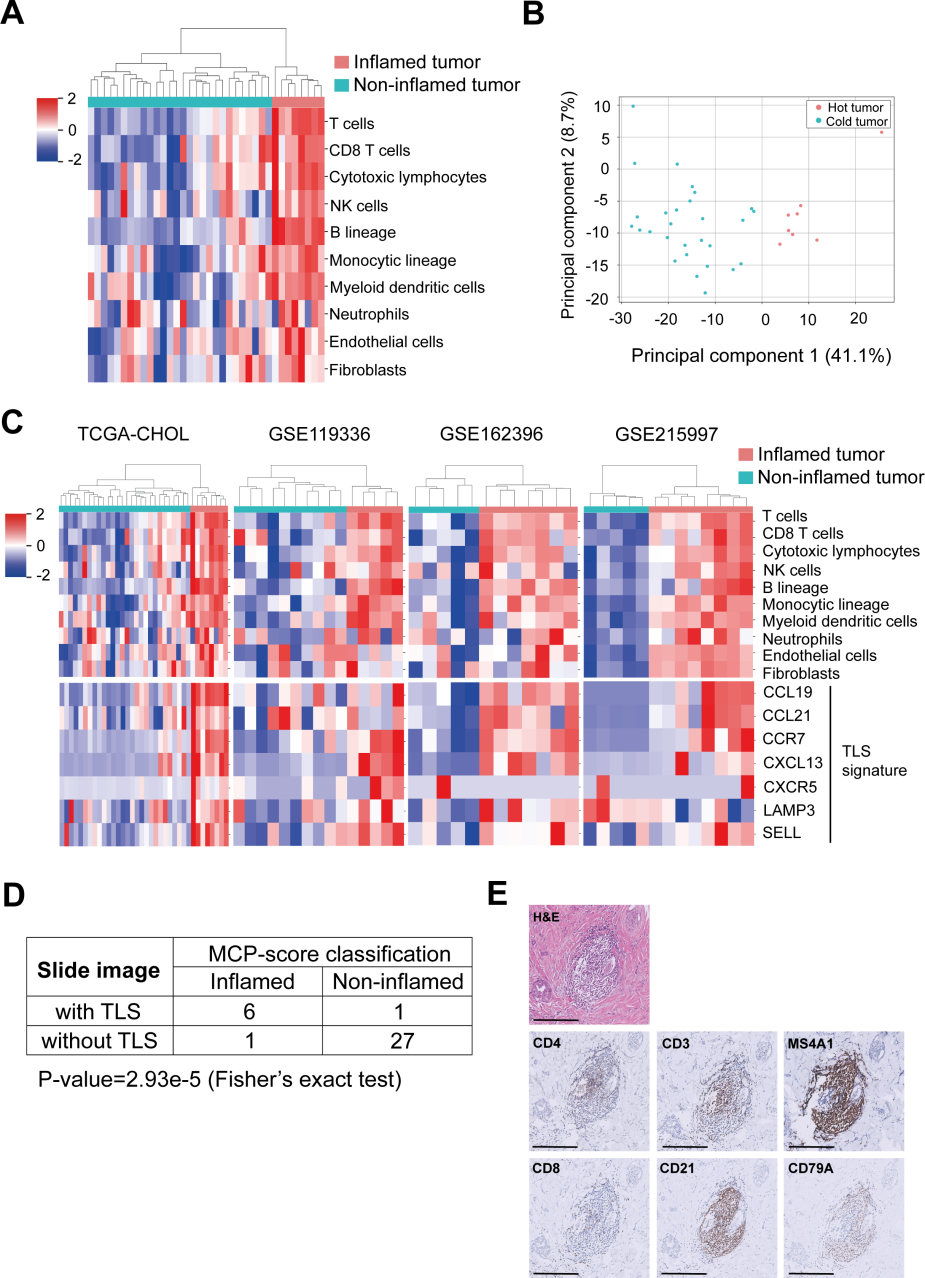
Correlations of inflamed tumors with TLS signatures. (**A**) Heatmap and hierarchical clustering revealed the abundance of 10 cell types in inflamed and non-inflamed tumor samples derived from the TCGA-CHOL data set. (**B**) Principal component analysis performed using 109 transcriptomic markers (TCGA-CHOL data set) indicated that inflamed and non-inflamed tumors can be differentiated by principal component 1. (**C**) Heatmap and hierarchical clustering showed the abundance and clusters of 10 cell types (upper panel) and the expression of DC-LAMP (dendritic cell-lysosomal-associated membrane protein), CCR7 (C-C chemokine receptor 7), CCL21 (C-C motif chemokine ligand 21), CXCL13 (C-X-C motif chemokine ligand 13), CXCR5 (C-X-C chemokine receptor type 5), CCL19 (C-C motif chemokine ligand 19), and SELL (L-selectin) with the cluster of different groups (lower panel); relevant data were derived from the TCGA-CHOL, GSE119336, GSE162396, and GSE215997 data sets. (**D**) Confusion matrix comparing inflamed and non-inflamed tumors with TLSs (detected through whole-slide image analysis); relevant data were derived from the TCGA-CHOL data set. (**E**) Results of H&E staining and immunostaining (CD4, CD8, CD3, CD21, MS4A1, and CD79A) for our clinical sample. Magnification, 200x. TCGA, The Cancer Genome Atlas; TLS, tertiary lymphoid structure.

Cholangiocarcinoma tumors have traditionally been considered to exhibit low immune infiltration and limited responses to immune checkpoint inhibitors (ICIs).^32 33^ To study the complex TME, we analyzed the RNA-Seq data of 36 cholangiocarcinoma samples (derived from the TCGA-CHOL data set) by using MCP-counter.^29^ On the basis of MCP score heatmaps and hierarchical clustering results, the samples were classified as inflamed or non-inflamed tumors. Substantial immune infiltration was observed in inflamed tumors (figure 2A). Similar findings were obtained for the samples derived from the GSE119336, GSE162396, and GSE215997 data sets, all of which consist of ICC cases. These datasets include 15 samples from China and 25 samples from South Korea (12 and 13 samples, respectively). These findings indicate the presence of inflamed tumors in patients with cholangiocarcinoma (figure 2C).

To identify pivotal genes and immune cells within inflamed and non-inflamed tumors, we conducted a PCA using 109 transcriptomic markers derived from the MCP score calculations, enabling the comprehensive characterization of molecular and immune landscape differences between the two tumor subtypes. The analysis confirmed that the expression patterns of these 109 genes, represented by principal component 1 (PC1), effectively distinguished between inflamed and non-inflamed tumors (figure 2B), underscoring the potential of these gene signatures as reliable biomarkers for tumor classification. Thus, we ranked these genes on the basis of their weighting coefficients. The list of top 10 of 109 genes indicated the substantial upregulation of genes related to T cells and B-cell lineages, particularly a subset of B cells (table 1). Collectively, the results validated the presence of inflamed tumors in patients with cholangiocarcinoma. In various cancers, the tumor mutation burden serves as a key determinant of the tumor response to immunotherapy.^34 35^ The tumor mutation burden can also indicate the status of immune infiltration across tumor regions.^34^ Thus, using data from the TCGA-CHOL data set, we investigated the tumor mutation burden in inflamed and non-inflamed tumors (online supplemental figure 1). Surprisingly, no significant difference was noted in the tumor mutation burden between inflamed and non-inflamed tumors. Therefore, compared with other cancers, distinct approaches can be used for differentiating inflamed tumors from non-inflamed tumors in cholangiocarcinoma.

**Table 1:** Top 10 differentially expressed genes between inflamed and non-inflamed tumors

Entrez ID	931	973	930	79 368	3514	1380	5079	55 024	29 851	50 852
Gene symbol	MS4A1	CD79A	CD19	FCRL2	IGKC	CR2	PAX5	BANK1	ICOS	TRAT1
Cell type	B lineage	B lineage	B lineage	B lineage	B lineage	B lineage	B lineage	B lineage	T cells	T cells

### Tlss and inflamed tumors

Cytotoxic T cells play crucial roles in immunotherapy by activating and regulating the immune system to fight pathogens or foreign antigens.^36^ In the context of immune responses, the effects of TLSs on the TME remain poorly understood, which prompted us to conduct the present study. We identified well-known TLS signatures from the literature.^27^ Analysis of the TCGA-CHOL and GEO datasets demonstrated a marked overexpression of TLS-associated gene signatures in inflamed tumors (figure 2C). To further explore this association, we reviewed whole-slide images from the TCGA-CHOL cohort and identified a significant correlation between the presence of TLSs and inflamed tumor phenotypes (figure 2D). Additionally, immunohistochemical analysis of cholangiocarcinoma samples from our clinical study confirmed TLS positivity, characterized by MS4A1+follicular B cells surrounded by CD3+T cells (figure 2E). In parallel, multiplex IHC conducted on the same patient’s specimens which coexistence of TLS and TIL, demonstrating that TLSs exhibited a higher density of B cells (CD20) and an increased expression of T cells (CD3, CD4, CD8) compared with TIL (online supplemental figure 2). Our findings suggest that TLSs exhibit features commonly associated with inflamed tumors in cholangiocarcinoma.

### Gene expression in TILs and TLSs

**Figure 3:**
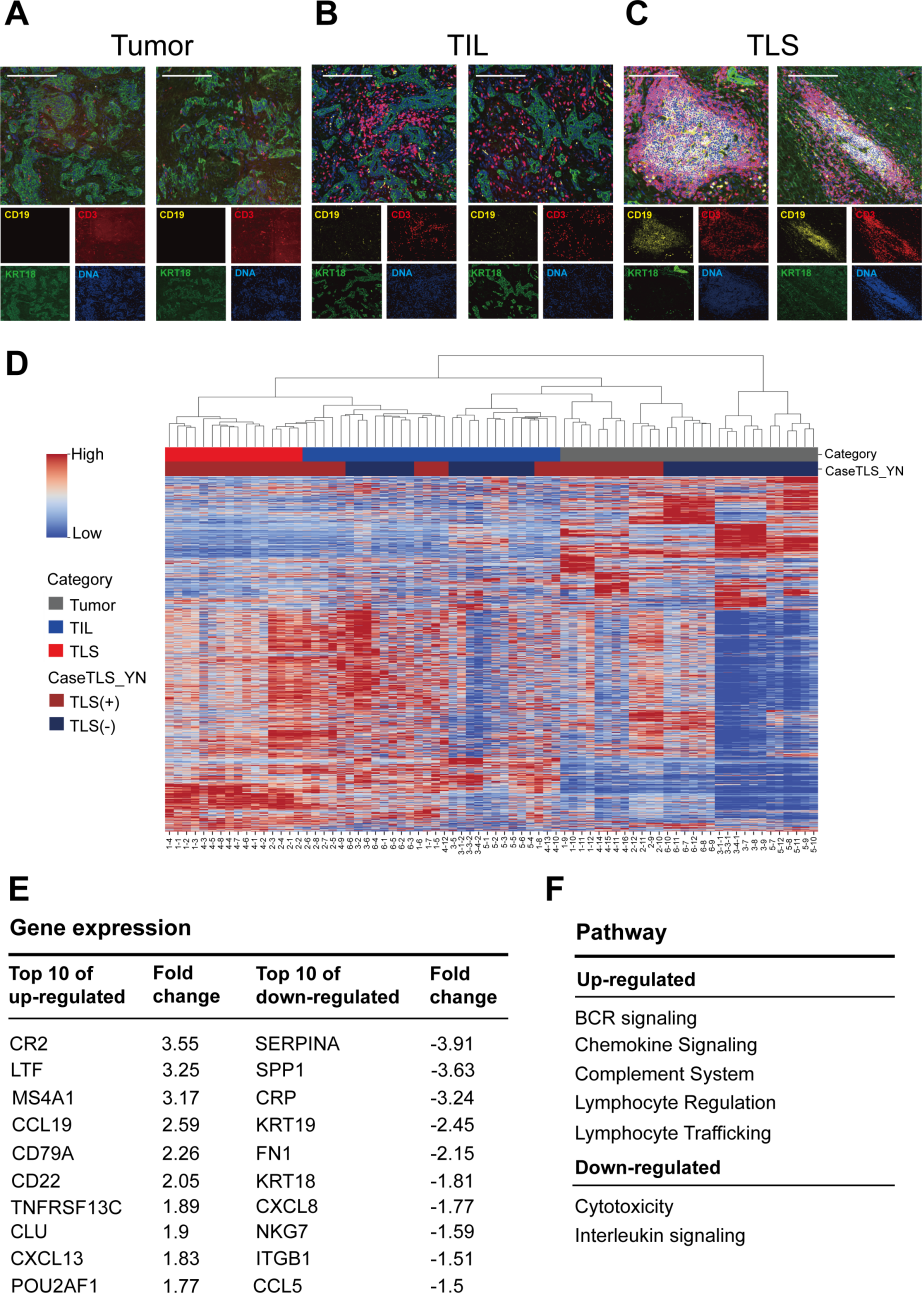
Gene expression in tumors, TILs, and TLSs. (**A**) Tumor, (**B**) TILs, and (**C**) TLSs in human cholangiocarcinoma samples. Tumor tissues were stained with antibodies against CD19 (yellow), CD3E (red), KRT18 (green), or DNA (blue). Magnification, ×20. (**D**) Heatmap and hierarchical clustering indicating gene expression profiles and clusters for tumors, TILs, and TLSs. (**E**) Top 10 differentially expressed (upregulated or downregulated) genes between TLSs and TILs. (**F**) Pathways involving the upregulated or downregulated genes. TIL, tumor-infiltrating lymphocyte; TLS, tertiary lymphoid structure.

To further characterize the features of TILs and TLSs within the TME, we analyzed six cholangiocarcinoma samples from our study. Base on H&E staining, the samples were stratified into TLS-positive (n=3) and TLS-negative samples (n=3). Following this stratification, the samples were subjected to analysis using GeoMx DSP technology for a detailed molecular and spatial assessment. ROIs were defined using four fluorescent markers to precisely distinguish key components of the TME. Each ROI contained a single AOI, ensuring focused analysis of specific regions. KRT18 antibodies (green) were used to differentiate between tumorous and stromal compartments, whereas CD3E (red) and CD19 (yellow) antibodies were used to detect T- and B-cell markers, respectively. In addition, 4′,6-diamidino-2-phenylindole (DAPI) staining (blue) was performed to identify cell nuclei. The organization of tumors, TILs, and TLSs is depicted in figure 3A–C. In tumors with fewer CD19^+^ and CD3^+^ cells, diffuse CD19 and CD3 expressions were observed around tumor cells, indicating the presence of TILs. By contrast, TLSs exhibited the concentrated aggregation of CD19^+^ and CD3^+^ cells, which facilitated the identification of TLSs. Based on these immunological markers, we classified all ROIs (n=72) into tumors (n=30), TILs (n=30), and TLSs (n=12).

We analyzed the expression of 1834 genes, derived from the GeoMx CTA, a panel specifically designed for comprehensive profiling of tumor biology, the TME, and the immune response, to study differential gene signatures between tumors, TILs, and TLSs. The resulting heatmap revealed distinct gene expression profiles between these regions, with notable differences observed between tumors and both TILs and TLSs (figure 3D). To compare TIL and TLS signatures from the 1834 genes, we conducted an analysis of differentially expressed genes (DEGs), focusing on those with significant upregulation or downregulation (log-2 fold change >1; p<0.05; figure 3E). The top 10 differentially expressed (upregulated) genes between TLSs and TILs were related to B cells. Among these genes, *MS4A1*, *CD79A*, *TNFRSF13C*, and *POU2AF1* are essential for the activation, maturation, and survival of B cells. Additionally, CR2, also known as CD21, is a receptor that is expressed on B cells and follicular DCs. CCL19 and CXCL13 are key chemokines that recruit lymphocytes into TLSs and facilitate the formation of TLS.^37 38^ Notably, TLSs exhibited the overexpression of MS4A1, CD79A, and CR2, as revealed by the analysis of the TCGA data (table 1). Comparative pathway analysis between TILs and TLSs revealed elevated levels of B-cell receptor signaling, complement activation, and lymphocyte regulation and trafficking, while cytotoxic activity was reduced in TLSs compared with TILs (figure 3F). Additionally, we were interested in comparing TILs and TLSs from the same samples in a paired analysis. We selected three targets comprizing TIL (n=4) and TLS (n=4) sites. The samples were pooled and normalized, resulting in a total of 12 TILs and 12 TLSs for comparison. After conducting statistical analysis, we identified 364 DEGs, which were used for hierarchical clustering and bioinformatic predictions. The hierarchical clustering heat map revealed unique expression patterns, from which we extracted the DEG signature (online supplemental figure 3). This signature predicted diverse signaling pathways, including those related to immune responses and oncogenesis. Our analysis identified that cytotoxic T lymphocytes and several cytokines were more activated in the TIL group compared with the TLS group. Conversely, B lymphocytes and B cell receptor signaling were suppressed in the TIL group, suggesting that the TLS region harbored a higher proportion of B cells, consistent with our previous hypothesis (online supplemental table 1). Additionally, we identified several transcription factors involved in the TME. Based on previous studies, TBX21 (T-bet), PRDM1, and TOX have been reported to be transcriptionally regulated in neoantigen-specific TILs in cancer.^39^ Conversely, transcription factors such as PAX5 and IKZF1, which are implicated in promoting TLS formation or reducing TILs, were also consistent with our predictions^40 41^ (online supplemental table 2). Among these findings, we highlight several aspects that merit further investigation.

### Immune classification of TILs and TLSs

**Figure 4:**
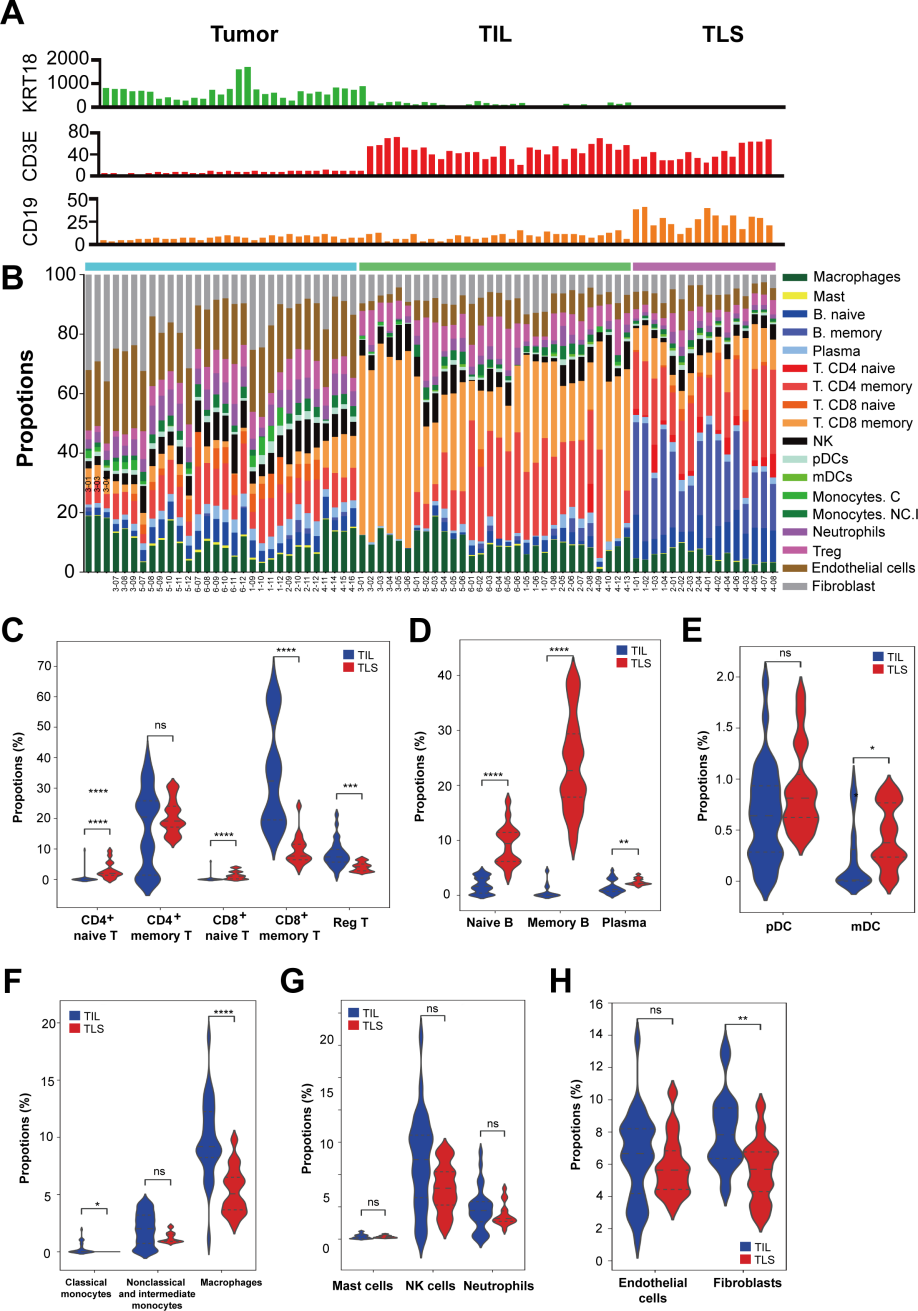
Immune cell compositions of tumors, TILs and TLSs. (**A**) Cellular composition of selected ROIs, showing KRT18 (tumor marker), CD3E (T cell marker), and CD19 (B cell marker) expression. (**B**) Pair-wise comparison of immune cell composition between tumors, TILs, and TLSs in cholangiocarcinoma samples. Properties of (**C**) T cells, (**D**) B cells, (**E**) dendritic cells, (**F**) macrophages, (**G**) innate cells, and (**H**) endothelial cells and fibroblasts. *p<0.05, **p<0.01, ***p<0.005, ****p<0.001, Mann-Whitney U test. ROIs, regions of interest; TIL, tumor-infiltrating lymphocyte; TLS, tertiary lymphoid structure.

To characterize the signatures of immune cell populations in TILs and TLSs, we estimated the properties of 18 types of immune cells, including T cells, B cells, NK cells, DCs, macrophages, neutrophils, mast cells, fibroblasts, and endothelial cells in each ROI (figure 4B) using SpatialDecon, which combines gene expression deconvolution with scRNA-seq data to estimate the spatial arrangement and abundance of immune cells in tissue samples and further validated against marker proteins to ensure accuracy.^42^ Our results revealed that TLSs exhibited higher properties of naive T cells, including CD4^+^ and CD8^+^ T cells, compared with TILs. In contrast, TILs had abundant CD8^+^ memory T cells and regulatory T cells. Notably, the predominant signature of T cells in TLSs were memory CD4^+^ T cells (figure 4C), which aligns with previous findings showing elevated proportions of CD4^+^ memory and naïve T cells in TLSs compared with the surrounding tumor tissue.^43^ We also observed elevated properties of B-cell lineages, including naive, memory, and plasma cells, with TLSs showing particularly high levels of memory B cells (figure 4D). Given that the primary function of DCs is to facilitate T-cell infiltration through the activation of naive T cells,^21 44^ it is noteworthy that myeloid DCs were more abundant in TLSs than in TILs (figure 4E). However, the properties of various innate immune cells, such as macrophages, NK cells, mast cells, and neutrophils, were lower in TLSs than in TILs (figure 4F–H). Collectively, our findings suggest that compared with TILs, TLSs exhibit elevated properties of naive T cells, B cells, and myeloid DCs but reduced properties of innate immune cells.

### TLSs influence T-cell phenotypes and immune checkpoint molecules in the TME

**Figure 5:**
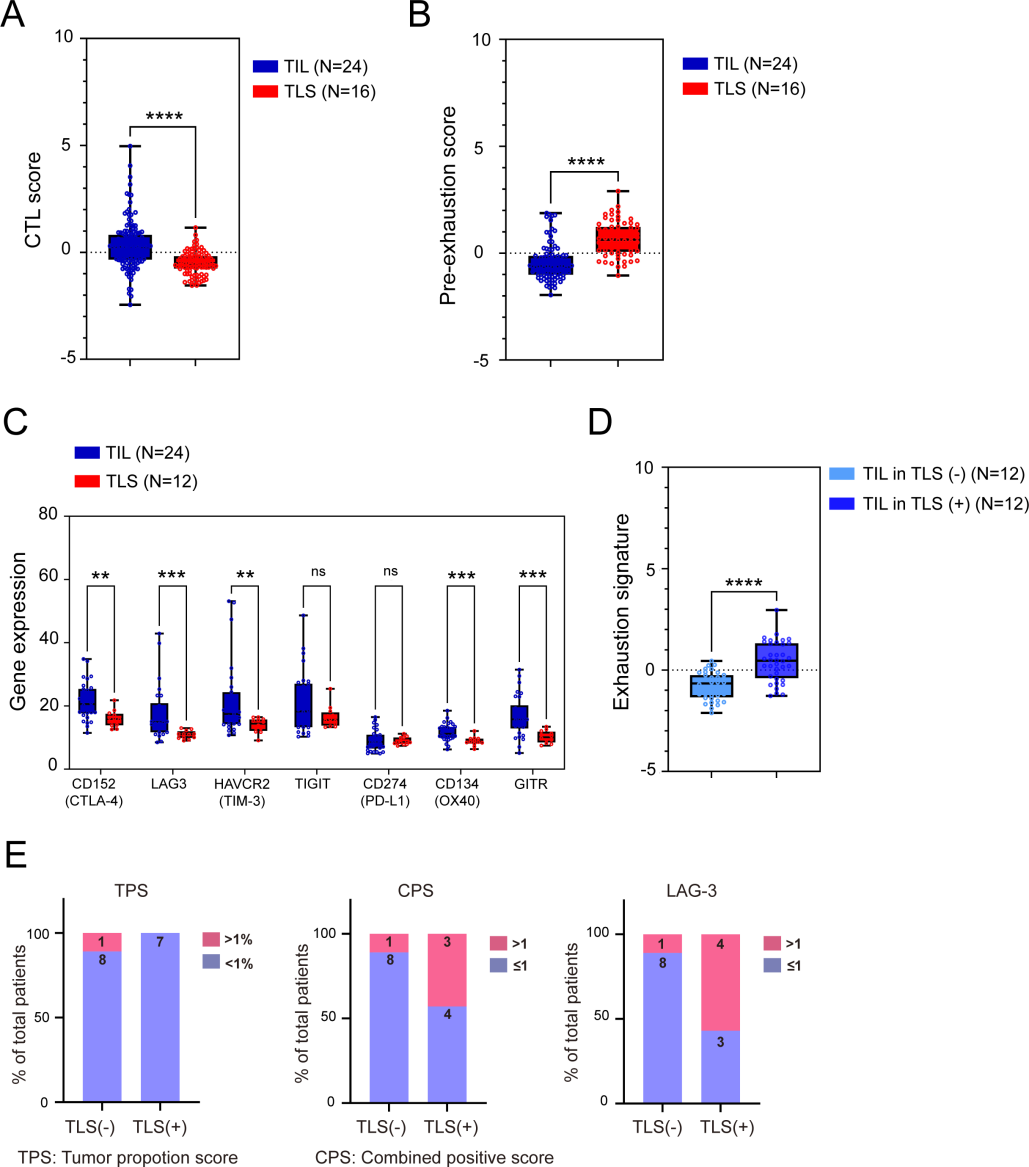
T-cell profiles, immune checkpoint molecules, and TLSs influence the cholangiocarcinoma microenvironment. Heatmap depicting the signature of (**A**) cytotoxicity and (**B**) precursor-exhausted T cells in TILs or TLS tumors. (**C**) Levels of immune checkpoint molecules in TILs and TLSs. Heatmap depicting the signature of (**D**) exhausted T cells in TIL within TLS-positive or TLS-negative. (**E**) Proportions of TLS-positive or TLS-negative patients with tumor proportion scores of ≥1% or <1% and combined positive scores of ≥1 or <1. The assessments were performed through PD-L1 staining (22C3) and LAG-3 staining of cholangiocarcinoma samples. Corresponding p values (Fisher’s exact test) were 0.0007,<0.0001, and <0.0001, respectively. **p<0.01, ***p<0.005,****p<0.001, Mann-Whitney U test. TIL, tumor-infiltrating lymphocyte; TLS, tertiary lymphoid structure.

Due to the limited number of clinical samples, it was challenging to validate all proteins comprehensively. Therefore, we used RNA sequencing data from GeoDSP and applied statistical scoring based on the gene signatures of specific T-cell subtypes to assess the differences between TLSs and TILs (figure 5A, B,D), as well as between TILs in TLS-positive tumors and those in TLS-negative tumors (figure 5C). This gene expression-based scoring method effectively captures the characteristics and activity of different T-cell subtypes, compensating for the limitations of protein validation and providing reference data to further elucidate the functional roles of T cells in various TMEs. The signature of CTL, precursor-exhausted, and exhaustion are calculated by relative genes expression. The signature of CTL includes the z-score expression of GZMA, GZMB, TBX21, CX3CR1, GNLY, and PRF1.^45^,^49^ The signature of precursor-exhausted consists of the z-score expression of IL7R, LTB, CCR7 and TCF7.^27 50 51^ The signature of exhausted includes the z-score expression of EOMES, GZMK and CD27.^52^,^56^ Our results revealed that TLSs exhibited decreased expression of CTL-related genes and elevated expression of precursor-exhausted-related genes signature, implying that the T cells surrounding TLS exhibit high potential of mouldability instead of cytotoxicity (figure 5A,B). In the meantime, we also analyzed immune checkpoint molecules to examine the immunity function difference between T cells in TIL and TLS. Obviously, TILs exhibited significantly elevated levels of the following immune checkpoint molecules such as CTLA4, LAG3, TIM-3, OX40, and GITR (figure 5C). Additionally, we dissected the expression profiles of exhaustion-related genes that differed between TILs in TLS-positive and TLS-negative tumors. Interestingly, the signature of exhausted is higher in TIL in TLS-positive tumors than TIL in TLS-negative tumors, indicating that those precursor-exhausted T cells displayed in TLS have a high chance of being educated to prefer T cells with exhaustion characteristics (figure 5D). This evidence suggests that the T-cell subtypes within TILs of TLS-positive patients exhibit characteristics of exhausted T cells, facilitating a favorable immune environment that could enhance cancer immunity and be more responsive to immunotherapy.

To further explore the pathways and regulatory elements potentially implicated in TME activation, we performed a detailed comparative analysis of tumors and TILs, both in the presence and absence of TLSs. DEGs were analyzed using Ingenuity Pathway Analysis to identify relevant molecular pathways and regulators. Given our focus on understanding the mechanisms by which TLSs mediate enhanced responses to immunotherapy, we specifically examined the regulators of PD-L1 expressions (online supplemental table 3). Our analysis revealed activation of the HIF transcription factor family in TILs and tumors associated with TLSs. We hypothesize that HIF-2 may regulate PD-L1 expression, potentially facilitating immune evasion mediated by TLSs in the TME.

To validate the findings from our transcriptome analyses, we analyzed real-world clinical data from the T1219 clinical trial (figure 5E). Tumor proportion scores (TPS), which were calculated in terms of PD-L1 expression levels in tumors, did not differ between TLS-positive patients and TLS-negative patients. However, the proportion of patients with a combined positive score (CPS) of >1 was significantly higher in the TLS-positive group than in the TLS-negative group (43% vs 11%, respectively; p=0.007). Similar trends were obtained through immunohistochemical staining for LAG-3. These results suggest that the TME in TLS-positive patients is conducive to favorable immunotherapy responses, with PD-L1 being expressed primarily in the TME rather than on the tumor cells themselves.

## Discussion

Following liver cancer, cholangiocarcinoma is the second most common malignancy of the hepatobiliary system, and it originates from the epithelial cells of the bile ducts. Because of a delay in symptom detection, surgical intervention may not be appropriate for all patients with biliary tract cancer. Currently, the standard treatment approach for cholangiocarcinoma involves combined immunotherapy and chemotherapy. However, not all patients benefit from this treatment. Moreover, few biomarkers have been identified for predicting the treatment response in patients with cholangiocarcinoma.

We explored the hitherto unexplored domain of TLSs in cholangiocarcinoma to evaluate their potential as predictive biomarkers and their effects on the TME. The analysis of data from patients with cholangiocarcinoma receiving nivolumab plus modified gemcitabine and S-1 therapy revealed the potential of TLSs as predictive biomarkers. The predictive potential was evident from the markedly improved treatment response rates, enhanced progression-free survival, and prolonged overall survival in TLS-positive patients (figure 1). The classification of cholangiocarcinoma samples into inflamed or non-inflamed tumors revealed the distinct molecular and cellular landscapes of these type types of tumors. Notably, the upregulation of B-cell lineage-related genes delineated inflamed and non-inflamed tumors, highlighting the heterogeneity of cholangiocarcinoma samples (figure 2; table 1). Whole-slide images and TLS signatures indicated that TLSs share characteristics with inflamed tumors, emphasizing their potential for predicting immunotherapy responses.

**Figure 6:**
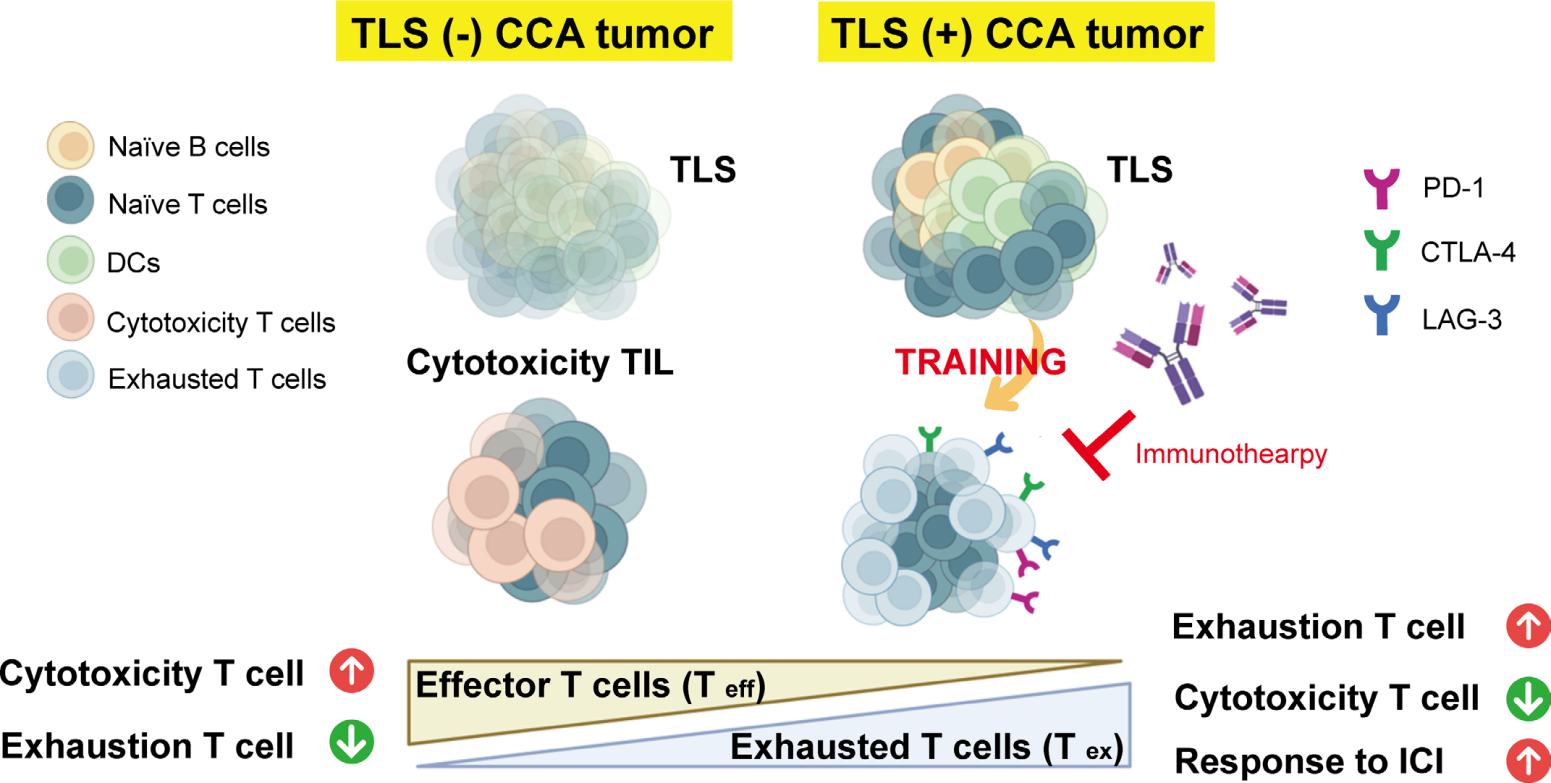
Schematic indicating the potential of TLSs in improving immunotherapy responses in patients with cholangiocarcinoma. TLSs increased the signature of exhausted T cells (by drawing from surrounding tumor cells) to evade immune surveillance. CCA, cholangiocarcinoma; DC, dendritic cell; ICI, immune checkpoint inhibitor; TLS, tertiary lymphoid structure.

Spatial analyses revealed the gene expression profiles of tumors, TILs, and TLSs in cholangiocarcinoma samples. Our results provided the locations, compositions, and differential gene expression patterns of tumors, TILs, and TLSs in the samples. Specifically, we observed high levels of B-cell activation-related signatures in TLSs; by contrast, high proportions of cytotoxic T cells were noted in TILs (figure 3). Our results are similar to previous study that most ICI-responsive B cells are a subset of memory B cells.^57^ Furthermore, an improved ICI response is observed in patients harboring immunoglobulin (Ig)G-positive tumors. This enhancement is attributed to the role of TLSs in facilitating the in situ maturation of B cells, generating IgG^+^ and IgA^+^ plasma cells that disseminate throughout the tumor tissue along fibroblastic tracks.^58^ These findings underscore the importance of B cells, particularly memory B cells within TLSs, in the TME. Most importantly, TILs in TLS-positive tumors had higher signature of exhaustion markers compared with TILs in TLS-negative tumors (figure 4). Our findings are corroborated by those of a clinical trial reporting improved clinical outcomes in TLS-positive patients, particularly those harboring exhausted T cells, characterized by the coexpression of *PD1*, *LAG3*, *TIGIT*, and *TIM3*.^59^ The TPSs were similar in TLS-positive patients and TLS-negative patients. However, between-group differences were observed in the CPS and LAG3 expression level. This suggests that TLS-positive tumors may not naturally exhibit PD-L1 expression but impact the TME, ultimately leading to better responses to immunotherapy (figure 5). Our results confirm that TLSs in cholangiocarcinoma tumors can induce the transformation of surrounding precursor-exhausted T cells into T cells with exhausted cell properties, which then serve as exhausted T cells against tumors (figure 6). Collectively, these findings indicate that further next-generation immunotherapy research on the TME of TLS-positive tumors is warranted.

This study has some limitations in clinical application and unclear underlying mechanisms. In clinical practice, obtaining tissue samples from patients with cholangiocarcinoma is challenging. Another key challenge is the need for large tissue instead of biopsy samples to detect the presence of TLSs. These challenges reduce the number of clinical samples available for research and reduce the possibility that the presence of TLS can be directly observed clinically. Nevertheless, our findings align with those of two extensive clinical studies.^60 61^ Shang *et al* examined 100 patients with cholangiocarcinoma who received immunotherapy (cohort 2), demonstrating the potential of TLSs for predicting the outcomes of cholangiocarcinoma.^61^ On the other hand, our evidence suggests that TLS-positive patients exhibit more favorable outcomes following immunotherapy. This observation may be attributed to the higher prevalence of exhausted T cells within the TILs of TLS-positive patients, rendering them more amenable to immunotherapeutic interventions. However, the underlying mechanism for the higher prevalence of exhausted T cells within the TILs of TLS-positive CCA remains to be elucidated and warrants further investigation in future studies.

In conclusion, we elucidated the multifaceted role of TLSs in cholangiocarcinoma, offering insights into their predictive value and their effects on immune cell phenotypes and regulatory pathways within the TME. A comprehensive understanding of these complex interactions can facilitate the precise application of immunotherapy in patients with cholangiocarcinoma.

## supplementary material

10.1136/jitc-2024-010173 online supplemental file 1

## Data Availability

Data are available on reasonable request.
