# Supplementary material for: Comparative impact of tertiary lymphoid structures and tumor-infiltrating lymphocytes in cholangiocarcinoma
Source: J Immunother Cancer. 2025 Jan 27;13(1):e010173. doi: 10.1136/jitc-2024-010173 (PMC11772930; doi:10.1136/jitc-2024-010173)
Supplement: online supplemental file 1 [file jitc-13-1-s001.docx]

**
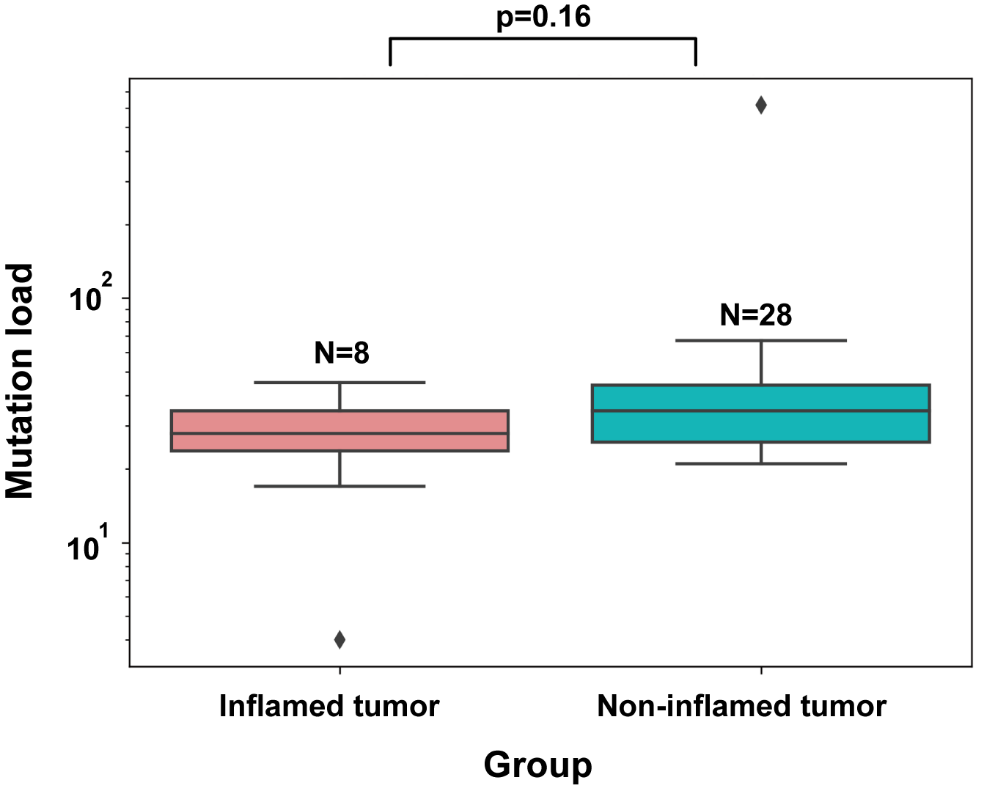
**

**Supplementary Figure 1. Categorization of inflamed and non-inflamed tumors across data sets analyzed in this study.**

(A) Heatmap and hierarchical clustering showing the abundance of 10 cell types (analyzed using MCP-counter) in inflamed and non-inflamed tumors. Relevant data were collected from three GEO data sets: GSE162396, GSE215997, and GSE119336. (B) Tumor mutation burden of inflamed and non-inflamed tumors. Relevant data were collected from the TCGA-CHOL data set.


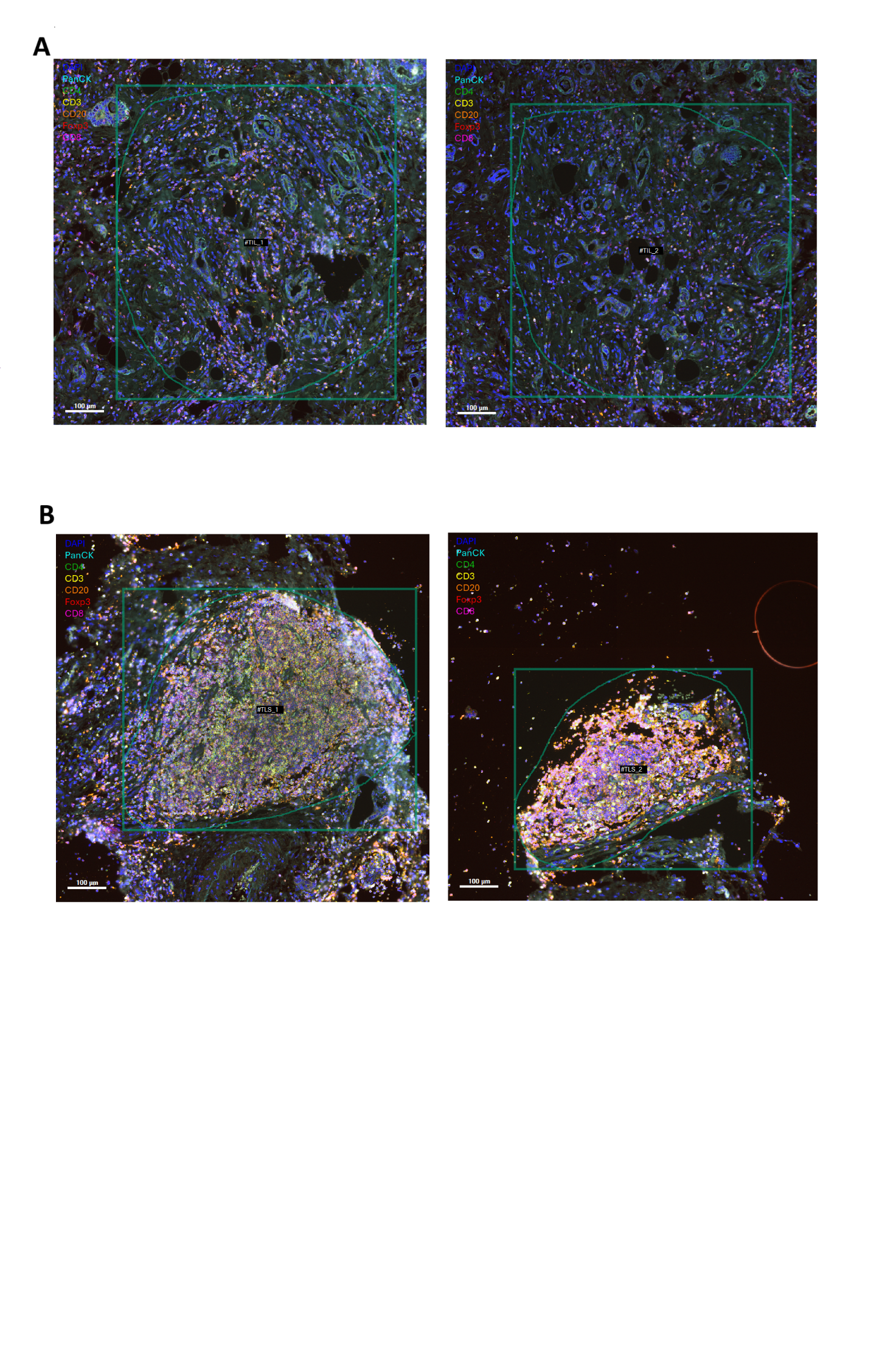


**Supplementary Figure 2**. **Multiplex Immunohistochemistry (IHC) Analysis of TIL and TLS in the same patient.**

Representative multiplex IHC image depicting the distribution and composition of (A) TIL and (B) TLS within the tumor microenvironment. Key markers include PanCK, CD3, CD4, CD8, CD20, and FOXP3. The scale bar is 100 μm.


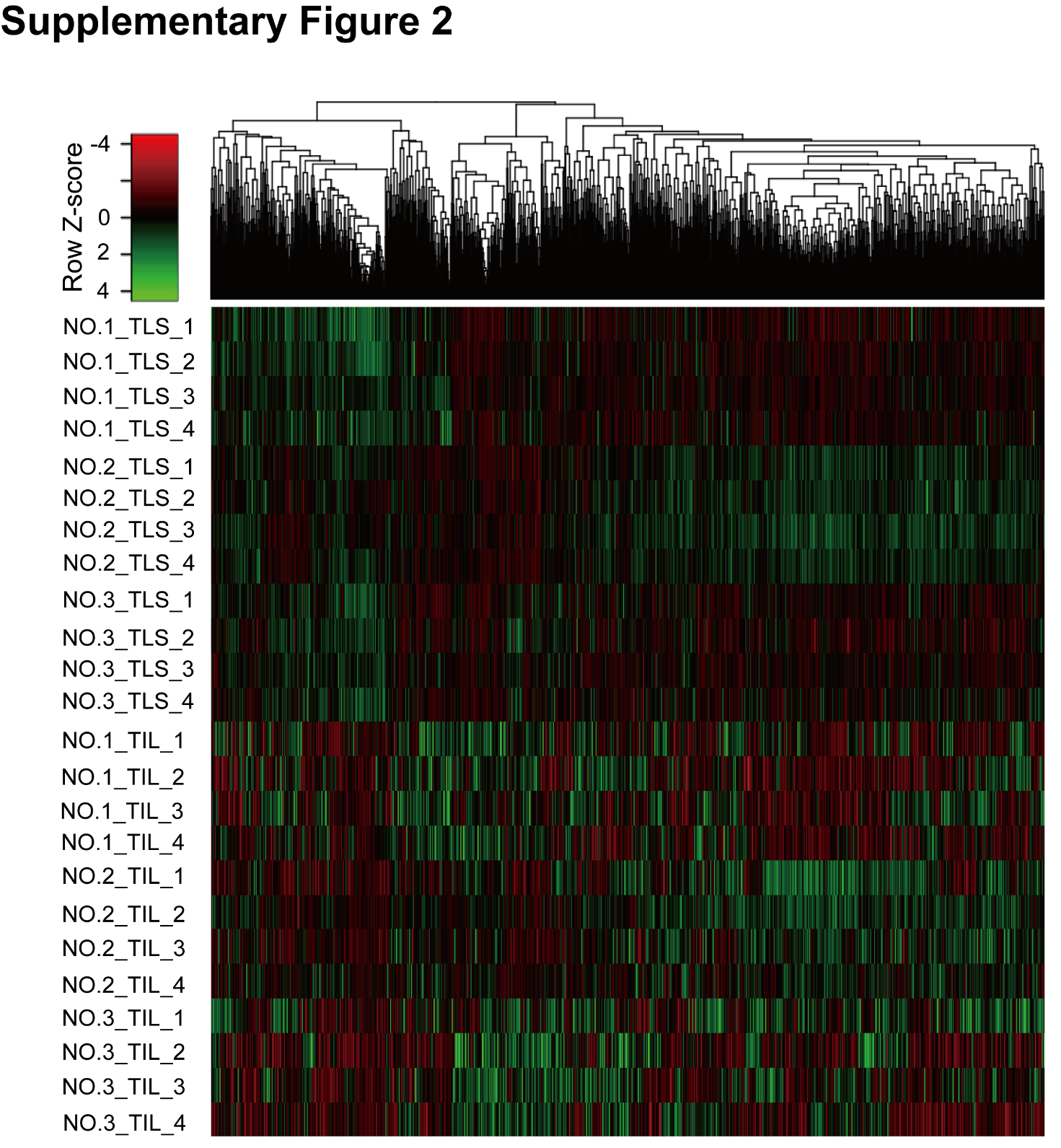


**Supplementary Figure 3**. **Hierarchy clustering plot performs the selection signature between TIL v.s. TLS in the same samples.**

**Supplementary Table 1. Prediction of potential canonical pathways.**

| **Ingenuity Canonical Pathways** | **-log(p-value)** | **ratio** | **z-score** |
| --- | --- | --- | --- |
| PI3K Signaling in B Lymphocytes | 7.7 | 0.0492 | -4.315 |
| B Cell Receptor Signaling | 9.3 | 0.0521 | -4.271 |
| Signaling by the B Cell Receptor (BCR) | 12 | 0.118 | -4.025 |
| Protein Kinase A Signaling | 5.98 | 0.0511 | -3.742 |
| p70S6K Signaling | 3.77 | 0.0363 | -3.71 |
| HIF1α Signaling | 14 | 0.115 | -3.545 |
| Gαq Signaling | 9.14 | 0.1 | -3.357 |
| BBSome Signaling Pathway | 0.945 | 0.0225 | 2.111 |
| Interleukin-10 signaling | 16.2 | 0.333 | 2.324 |
| CDC42 Signaling | 1.59 | 0.026 | 2.449 |
| CTLA4 Signaling in Cytotoxic T Lymphocytes | 9.77 | 0.0543 | 2.475 |
| IL-10 Signaling | 24.2 | 0.195 | 2.556 |
| Cytotoxic T Lymphocyte-mediated Apoptosis of Target Cells | 0.353 | 0.0165 | 2.646 |
| Endocannabinoid Cancer Inhibition Pathway | 11.1 | 0.122 | 2.668 |

**Supplementary Table 2. Prediction of potential upstream transcription factors by specific signature.**

| **Regulator** | **Expr Fold-change** | **Predicted** | **Activation z-score** | **p-value of overlap** |
| --- | --- | --- | --- | --- |
| PAX5 | -1.723 | Inhibited | -3.367 | 1.28e-20 |
| CIITA |  | Inhibited | -3.207 | 5.35e-16 |
| IKZF1 |  | Inhibited | -2.64 | 1.81e-25 |
| MLXIPL |  | Inhibited | -2.611 | 1.54e-03 |
| GATA6 |  | Inhibited | -2.38 | 3.47e-03 |
| EBF1 |  | Inhibited | -2.35 | 1.02e-19 |
| BCL6 |  | Inhibited | -2.342 | 1.06e-27 |
| IRF9 |  | Activated | 2.243 | 2.4e-07 |
| TP53 | -1.207 | Activated | 2.342 | 4.63e-32 |
| EBF4 |  | Activated | 2.639 | 1.02e-11 |
| BATF | 1.454 | Activated | 2.828 | 3e-09 |
| PRDM1 | 1.308 | Activated | 3.098 | 5.59e-37 |
| TBX21 | 1.334 | Activated | 3.123 | 2.36e-29 |
| ZBTB16 |  | Activated | 3.156 | 1.27e-31 |

**Supplementary Table 3**. **Candidate PD-L1 upstream regulators or transcription factors identified through ingenuity pathway analysis.** Pair-wise comparisons: TLSs versus tumors and TLSs versus TILs. Abbreviations: PD-L1, programmed death-ligand 1; TLS, tertiary lymphoid structure; TIL, tumor-infiltrating lymphocyte.

|  | **TIL in TLS+ v.s. TIL in TLS-** | **Tumor in TLS+ v.s. Tumor in TLS-** |
| --- | --- | --- |
| **PD-L1 Upstream Regulator** | **Activation z-score** | **Activation z-score** |
| TEAD1 | -0.886 | -0.359 |
| JUN | -1.791 | 2.218 |
| ZEB1 | 1.054 | 1.003 |
| NFKB1 | 1.064 | 4.395 |
| NFKB2 | 0.459 | 1.805 |
| MYC | 0.389 | 0.025 |
| STAT3 | 0.325 | 2.747 |
| LEF1 | -1.066 | -0.472 |
| IRF1 | 1.075 | 5.013 |
| IRF3 | 0.725 | 3.665 |
| HIF1A | 1.127 | 3.108 |
| EPAS1(HIF-2) | 1.864 | 2.172 |
